# Strong associations between national prevalence of various STIs suggests sexual network connectivity is a common underpinning risk factor

**DOI:** 10.1186/s12879-017-2794-x

**Published:** 2017-10-12

**Authors:** Chris Kenyon

**Affiliations:** 1 Sexually Transmitted Infections HIV/STI Unit, Institute of Tropical Medicine, Antwerp, Belgium; 2 Division of Infectious Diseases and HIV Medicine, University of Cape Town, Anzio Road, Observatory, 7700 South Africa

## Abstract

**Background:**

If national peak Human Immunodeficiency Virus (HIV) prevalence is positively associated with the prevalence of other sexually transmitted infections (STIs) from before or early on in the HIV epidemics this would suggest common underlying drivers.

**Methods:**

Pearson’s correlations were calculated between the prevalence of seven STIs at a country-level: chlamydia, gonorrhoea, trichomoniasis, syphilis, bacterial vaginosis, herpes simplex virus-2 (HSV-2) and HIV.

**Results:**

The prevalence of all the STIs was highest in the sub-Saharan African region excluding chlamydia. The prevalence of all seven STIs were positively correlated excluding chlamydia. The correlations were strongest for HIV-HSV-2 (*r* = 0.85, *P* < 0.0001) and HSV-2-trichomoniasis (*r* = 0.82, *P* < 0.0001).

**Conclusion:**

Our results of a generally positive association between the prevalences of a range of STIs suggests that higher prevalences were driven by common underlying determinants. We review different types of evidence which suggest that differential sexual connectivity is a plausible common determinant.

## Background

The spread of HIV around the world has been far from uniform. Only 20 countries have had generalized HIV epidemics (peak HIV prevalence greater than 5%) [1]. In these countries HIV prevalence typically increased from under 1% to over 10% in 10 years [2, 3]. Another 30 countries had peak HIV prevalence between 1 and 5% and in the remaining countries, HIV peaked below 1% [1]. Some authors have argued that this pattern of spread can be most parsimoniously explained by affected populations having more connected sexual networks [4–6]. Others have however argued that a range of other factors such as differences in the prevalence of circumcision, herpes simplex virus-2 (HSV-2) and other (sexually transmitted infections) STIs are responsible [7–10].

**Fig. 1:**
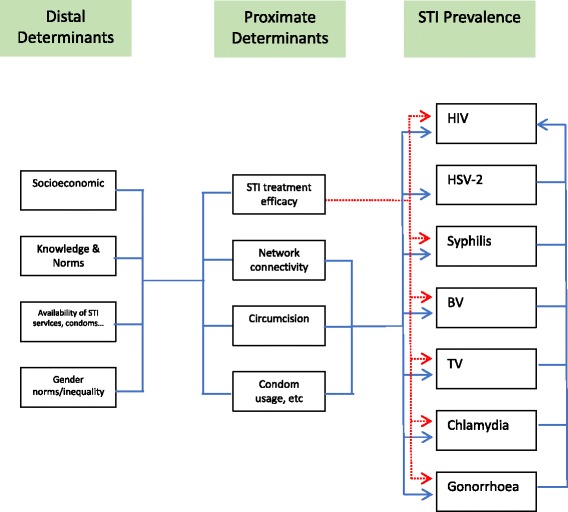
Conceptual framework to understand the relationship between proximate and distant determinants of HIV prevalence. Of note STI treatment efficacy can have an impact on the prevalence of all STIs except HSV-2 which is incurable (Dotted red arrows)

In this paper, we explore this issue from a novel angle by testing if there is an association between national peak HIV prevalence and the prevalence of other STIs from before or early on in the HIV epidemics. The rationale we use is that if peak HIV prevalence is strongly associated with other viral, bacterial and eukaryotic STIs then this suggests that a common factor underpins this. STIs like syphilis are curable and thus differences in syphilis prevalence may be due to differences in treatment efficacy. HSV-2, however, is incurable and thus treatment efficacy cannot explain differential HSV-2 spread (Fig. 1). If HSV-2 from early on in the HIV era is found to predict subsequent peak HIV prevalence this would suggest that the common risk factor is not STI treatment efficacy.

## Methods

Through preferentially affecting those at the highest risk parts of sexual networks the AIDS epidemics have been shown to reduce network connectivity and thereby STI transmission [11–13]. This effect has been noted in both men who have sex with men in the United States and in general populations in Africa [11, 12, 14]. To avoid the misclassification bias this could introduce, we used national STI prevalence figures from as early on in the HIV epidemics as were available in sufficient breadth and quality. As described in more detail below, these were generally from the year 1990 or before. We chose to relate these STI prevalence data to peak HIV prevalence rather than HIV prevalence estimates from the same time as the STI prevalence data as this has been shown to avoid the HIV introduction bias [2].

### *Chlamydia, gonorrhoea, syphilis,* trichomoniasis

Data for the prevalence estimates for gonorrhea, chlamydia, trichomoniasis and syphilis in the year 1990 were taken from the Global Burden of Diseases Study [15]. For this study the incidence and prevalence of each of these STIs were estimated by a literature and database review conducted by the World Health Organization. Prevalence refers to the percent of women 10 years of age or older who are infected with each STI at the beginning of 1990.The figures are age-standardized. The prevalence data used to model infection came from a comprehensive literature review conducted by a panel of STI epidemiology experts at the World Health Organization. Only studies sampling low risk subjects (primarily antenatal populations) were included. The data used spanned the years 1981 to 2010. The prevalence data and gaps therein were modelled separately using DisMod 3, a meta-regression tool that forces all parameters (prevalence, remission etc.) into consistency with one another [16].

### HSV-2

The HSV-2 prevalence data we used (prevalence in 40–44 year old women) was extracted from a previously published study that assessed the association between HSV-2 prevalence in 40–44 year old women and peak HIV prevalence [17]. The comparative HSV-2 prevalence data for this study was taken from two systematic reviews of global HSV-2 incidence and prevalence [18, 19]. These reviews were conducted in 2002 and 2005 and used data published between 1986 and 2003. Only studies that reported age and gender specific HSV-2 prevalence (assessed by type specific serology) and measuring this in population-based samples were included.

### Bacterial vaginosis (BV)

BV prevalence estimates were extracted from a systematic review of the global epidemiology of BV [20]. This study summarized BV prevalence from studies spanning the period 1984 to 2012. Only studies that used representative population samples or low risk antenatal samples were included. The Nugent scoring system has been recommended as the gold standard for studies comparing the prevalence of BV [21]. We therefore only included studies where the diagnosis of BV was based on the Nugent system. BV is generally regarded as a sexually associated disease rather than an STI but in the interests of succinctness we describe it as an STI.

### National peak HIV prevalence

National HIV prevalence estimates were taken from 1990 to 2009 in the Global Health Observatory Data Repository of the World Health Organization (http://apps.who.int/gho/data/node.main.622). These estimates are based on population-based testing, antenatal clinical surveillance, and epidemic models [22]. We used these data to calculate the peak HIV prevalence variable as the highest HIV prevalence (in 15- to 49-year-olds) attained in each country obtained between the years 1990 to 2009 (median year, 1998; interquartile range, 1996–2005) [1, 2].

### Ethical statement

The research involved secondary data analysis of STI prevalence estimates. No specific ethics committee approval was therefore necessary for this study.

### Statistical analysis

Pearson’s correlation (with Bonferroni corrections) was used to evaluate the relationship between each of the STIs. HIV prevalences were natural log- and TV prevalences cubic-transformed to create more normal distributions for regression analyses. All analyses were performed in STATA 13.0 (StataCorp LP, College Station, TX).

## Results

There were large variations in the national prevalences of HIV (median 0.4%, IQR 0.1–1.6), HSV-2 (median 27.4%, IQR 17.5–50.9), chlamydia (median 25.0/1000, IQR 21.6–42.1), gonorrhoea (median 9/1000, IQR 7.4–10.1), syphilis (median 0.1/1000, IQR 0.0–0.2), trichomoniasis (median 22.9/1000, IQR 18.8–26.4) and BV (median 18.3%, IQR 13.7–29.2; Table 1).

**Table 1:** Median (interquartile range) prevalence of seven sexually transmitted infections in all countries and those in sub Saharan Africa

	N^a^	All countries	N^a^	Sub Saharan Africa
HIV	170	0.4 (0.1–1.6)	51	3.5 (1–7.9)
HSV-2	64	27.2 (17.5–50.9)	20	64 (50.9–79.5)
Chlamydia	166	2.5 (2.2–3.4)	51	2.2(2.1–2.2)
Gonorrhea	166	0.9 (0.8–1.0)	51	1.1 (1.0–1.2)
Syphilis	166	0.01 (0.009–0.02)	51	0.02 (0.02–0.02)
BV	42	18.3 (13.7–29.2)	13	30.3 (25.0–38.1)
Trichomoniasis	166	2.3 (2.0–3.9)	51	4.1(4.0–4.2)

^a^ Number of countries with data for those with peak HIV prevalence data available

*Abbreviations*: *HIV* human immunodeficiency virus, *HSV-2* herpes simplex virus-2, *BV* bacterial vaginosis

**Fig. 2:**
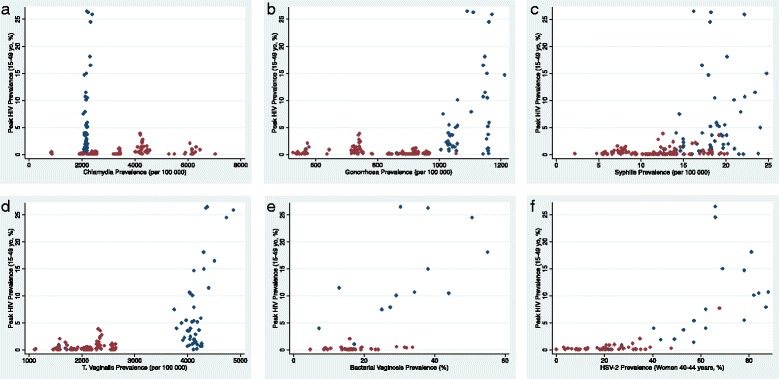
Association between national peak HIV prevalence and chlamydia (**a**), gonorrhea (**b**), syphilis (**c**), *T. vaginalis* (**d**), bacterial vaginosis (**e**), HSV-2 prevalence (**f**). (Blue dots - countries in sub Saharan Africa, red dots- other countries, data sources detailed in text)

The prevalence of all seven STIs were positively correlated excluding chlamydia which was negatively correlated with the other STIs (Table 2a, Fig. 2). The correlations were strongest for HIV-HSV-2 (*r* = 0.85, *P* < 0.0001) and HSV-2-trichomoniasis (*r* = 0.82, *P* < 0.0001). The prevalence of all the STIs was highest in the sub-Saharan African region (Table 1, Fig. 2) excluding chlamydia. The chlamydia prevalences for sub-Saharan African countries clustered tightly around 2000/100000 population. The positive correlations were largely driven by higher STI prevalences in sub Saharan Africa. Repeating the correlations limited to non-sub Saharan Africa reduced the strength of the associations (Table 2b).

**Table 2:** Pair-wise correlation between the prevalence of 7 sexually transmitted infections in all countries (a) and excluding countries from sub Saharan Africa (b) [Data from various sources]

	HIV	HSV-2	Chlamydia	Gonorrhoea	Syphilis	Trichomoniasis
a)						
HIV	–					
HSV-2	.85***	–				
Chlamydia	−.11	−.23	–			
Gonorrhoea	.44***	.58***	−.69***	–		
Syphilis	.50***	.67***	−.03	.15	–	
Trichomoniasis	.75***	.83***	−.41***	.75***	.56***	–
BV	.65***	.73***	−.22	.43	.50*	.60**
b)						
HIV	–					
HSV-2	.46*	–				
Chlamydia	.34**	.27	–			
Gonorrhoea	−.25	−.18	−.62***	–		
Syphilis	.07	−.02	.37***	−.60***	–	
Trichomoniasis	.10	.12	−.13	.48***	−.23	–
BV	.29	.29	−.05	.05	.26	.15

*P*-Value: * < .05, ** < .005, *** < .0005 (Including Bonferroni corrections)

*Abbreviations*: *HIV* human immunodeficiency virus, *HSV-2* herpes simplex virus-2, *BV* bacterial vaginosis

## Discussion

We found a strong positive association between 6 of the 7 STIs evaluated. Countries with high peak HIV prevalences had high prevalences of other STIs –excluding chlamydia- preceding or early in the HIV epidemics. This pattern strongly suggests that a common factor(s) underpinned the more extensive spread of STIs in these populations. To some extent these associations were driven by the higher STI prevalences in sub-Saharan Africa. The clear exception was chlamydia where the prevalence estimates for sub-Saharan Africa clustered tightly in the lower tertile of prevalence estimates. There are a number of possible explanations for the differences in distribution between chlamydia and other STIs. The fact that the prevalence estimates of chlamydia for African countries clustered tightly around a single value may be due to the low number of data points available for this region for this time point – 21 data points for the period 1981 to 2010 [15]. It was not specified how many of these data points were used to provide the estimates for the 1990 estimates. It is noteworthy that the WHO in its first global prevalence of STIs report in 1995 estimated the prevalence of chlamydia in sub Saharan Africa in 15–49 year old women and men was 7.1% and 4.8%, respectively – considerably higher than all other world regions [23, 24]. By the 2000 and 2005 global reports, the estimated prevalence of chlamydia in sub-Saharan Africa was lower than in a number of other regions. A combination of chlamydia’s long duration of (frequently asymptomatic) infection which enables it to spread easily in sexual networks and a degree of immunity following natural clearance of the infection may result in a higher prevalence of chlamydia in lower risk sexual networks (and lower prevalence of other STIs) than higher risk networks [25, 26].

### Limitations

The validity of our study depends on the accuracy of the individual STI prevalence estimates. Whilst the HIV estimates are based on high quality data (frequent nationally representative samples) the HSV-2 and BV estimates are based on intermediate quality data and the bacterial/protozoan STI estimates on modelled data of, at times, a low number of data points. Our results are however largely concordant with studies using different data sources. One study that only used syphilis prevalence data from antenatal populations for example found a similarly strong association between syphilis prevalence in the 1990s and peak HIV prevalence [27]. With the exception of chlamydia noted above, the WHO global STI epidemiology reports from 1995, 2001, 2005 and 2012 [28–31] all noted higher prevalences of the bacterial/protozoan STIs in the sub Saharan African region.

There is a paucity of nationally representative STI prevalence data preceding the HIV era. Arguably the best quality data comes from antenatal syphilis prevalence (ASP) surveys whose prevalence estimates have been shown to reasonably approximate that of the population at large and where testing has been widespread for over 50 years [32]. A study that used antenatal syphilis prevalence to describe the global epidemiology of syphilis over the past century found that countries in sub Saharan Africa had a less marked decline in ASP following the introduction of penicillin and that ASP settled at a considerably higher plateau here than elsewhere, including other low and middle income countries [33]. ASP in Southern and Eastern African countries from the pre-AIDS period plateaued at a median 10% until the HIV epidemic; interquartile range, 6.5–11.5% [33, 34]. No correlation was found between syphilis screening and treatment efficacy and ASP [33]. A review of syphilis epidemiology based on case reporting conducted by the WHO for the years 1945 to 1958, also found that the incidence of syphilis was highest in sub Saharan Africa [35].

Further support for the clustering of STIs at national/regional level comes from epidemiological studies of other STIs, such as lymphogranuloma venereum, donovanosis, human papilloma virus and chancroid, from the pre/early HIV period which found the prevalence in sub-Saharan Africa to be as high or higher than other world regions [36–38].

### Clustering of STIs at world regional and sub-national levels

The best quality evidence for the clustering of STIs, however, comes from sub national populations. Studies from Ethiopia, Kenya, South Africa, Uganda, the United Kingdom and the United States have found that ethnic groups with high HIV prevalences had higher prevalences of other STIs in the pre- or early-HIV period [5, 17, 20, 39, 40]. In the USA, for example, data from nationally representative samples established that the prevalences of HSV-2, chlamydia, gonorrhea, syphilis, and trichomoniasis were 3.2, 6.5, 16.2, 4.9 higher in non Hispanic blacks than non Hispanic whites [5]. Studies based on antenatal syphilis prevalence, serological testing of military recruits and case based surveillance demonstrated that this relative difference in syphilis prevalence has been present for at least 70 years [33, 41–44]. Likewise, HSV-2 prevalence estimates from nationally representative surveys found considerably higher prevalences in non Hispanic blacks than non Hispanic whites in the United States since the first survey in 1976 [45, 46]. Similarly, data from antenatal surveys and case surveillance in the 1930s to 1950s in South Africa showed that ethnic groups that went on to have high peak HIV prevalence had approximately 10 times higher syphilis prevalence from the 1930s until contemporary times than ethnic groups that had low peak HIV prevalence [33, 47, 48]. Rates of HSV-2/other STIs by ethnic group in South Africa, Uganda and Kenya followed a similar pattern [17, 49–51].

STIs have also been found to cluster at the level of world regions. Using partitioning according to medoids, one study found that there was sufficient clustering of 6 STIs to support classifying WHO world regions according to high, medium and low prevalence regions [26].

### How can we explain the clustering of STIs?

A striking feature of the clustering of STIs is that it includes a wide range of viral, prokaryotic, and eukaryotic organisms. This makes it less likely that differential genetic susceptibility is responsible for these different STI rates [5]. Differential STI treatment efficacy has long been argued to play a dominant role [52]. Whilst STI treatment efficacy is of crucial importance, differential access to treatment cannot explain the differential spread of incurable STIs such as HSV-2 [17]. Our finding that HSV-2 is predictive of peak HIV prevalence thus means that treatment efficacy cannot by itself explain the clustering of STIs. Furthermore, studies that have examined this in detail have found little or no association between treatment efficacy and STI prevalence [33, 53]. Likewise, although condom usage and circumcision are protective for the acquisition of STIs, there is little evidence that they explain the higher prevalence of STIs in sub-Saharan Africa [54, 55]. In South Africa for example where HIV prevalence varies by 40-fold between ethnic groups, the high prevalence groups have a three-fold higher circumcision and higher reported condom usage than the low HIV prevalence groups [56]. Whilst HIV was considered by many to be a disease of poverty, studies using nationally representative HIV-serolinked samples from 19 countries have demonstrated that HIV prevalence generally increases monotonically with wealth [57].

An emerging body of evidence suggests that combinations of the following factors determine STI prevalence: differential sexual network connectivity (determined by factors such as partner number, timing and concurrency) [5, 58, 59], factors that enhance the probability of transmission per contact (such as age gap and absence of circumcision or condom usage) [60, 61] and STI treatment efficacy. According to this conception, each population will have its own combination of these factors and this specific network transmission index (NTI) will play a large role in determining the prevalence of STIs [61]. Although there is still considerable debate on the topic, the network connectivity component of NTI has been shown to be a parsimonious explanation for both inter- and intra-national variations in STI prevalence [58, 59, 61, 62]. Markers of network connectivity, such as sexual partner concurrency, have been found to be positively correlated with the prevalences of HIV [6], HSV-2 [63], BV [64], syphilis [65] and trichomoniasis [61]. Furthermore, historical studies from South Africa suggest that differential network connectivity by ethnic group could explain the differences in syphilis prevalence since the 1930s [33, 66, 67].

## Conclusion

The finding that differences in STI prevalence by population have persisted for over 50 years requires further study but suggests that radical STI prevention will require strategies that target the underlying determinants of NTI. Uganda’s ‘Zero Grazing’ campaign, where individuals were encouraged to have only one sexual partner at a time, is one example of an intervention that made progress in this regard [68].

Our results of a positive association between a range of STIs provide further impetus to efforts that more accurately delineate and target the common underlying determinants of elevated STI prevalence.
